# In Silico Quantification of Intersubject Variability on Aerosol Deposition in the Oral Airway

**DOI:** 10.3390/pharmaceutics15010160

**Published:** 2023-01-03

**Authors:** Azadeh A. T. Borojeni, Wanjun Gu, Bahman Asgharian, Owen Price, Andrew P. Kuprat, Rajesh K. Singh, Sean Colby, Richard A. Corley, Chantal Darquenne

**Affiliations:** 1 Department of Medicine, University of California, San Diego, CA 92093-0623, USA; 2 Applied Research Associates, Arlington Division, Raleigh, NC 27615-2963, USA; 3 Pacific Northwest National Laboratory, Richland, WA 99352, USA; 4 Greek Creek Toxicokinetics Consulting, LLC, Boise, ID 83714, USA

**Keywords:** computational fluid dynamics (CFD), inertial impaction, laryngeal particle deposition, oropharyngeal deposition

## Abstract

The extrathoracic oral airway is not only a major mechanical barrier for pharmaceutical aerosols to reach the lung but also a major source of variability in lung deposition. Using computational fluid dynamics, deposition of 1–30 µm particles was predicted in 11 CT-based models of the oral airways of adults. Simulations were performed for mouth breathing during both inspiration and expiration at two steady-state flow rates representative of resting/nebulizer use (18 L/min) and of dry powder inhaler (DPI) use (45 L/min). Consistent with previous in vitro studies, there was a large intersubject variability in oral deposition. For an optimal size distribution of 1–5 µm for pharmaceutical aerosols, our data suggest that >75% of the inhaled aerosol is delivered to the intrathoracic lungs in most subjects when using a nebulizer but only in about half the subjects when using a DPI. There was no significant difference in oral deposition efficiency between inspiration and expiration, unlike subregional deposition, which shows significantly different patterns between the two breathing phases. These results highlight the need for incorporating a morphological variation of the upper airway in predictive models of aerosol deposition for accurate predictions of particle dosimetry in the intrathoracic region of the lung.

## 1. Introduction

The extrathoracic upper airway acts as the first line of defense to prevent inhaled toxicants from reaching the lungs but also as a barrier to the delivery of inhaled drugs. The proportion of aerosol deposited in the oral cavity and throat depends on the flow field and the size of inhaled particles [1]. The intricate anatomy of the upper airway produces complex flow patterns and particle trajectories and is thus also an important factor affecting aerosol deposition in the human respiratory system [2]. Several studies have shown that deposition in the throat is a major source of variability in lung deposition [3,4,5].

Aerosol deposition in the oral airway has been studied both computationally [2,6] and experimentally [7,8,9]. Previous in vitro work with steady flow rates presented empirical correlations for predicting oral deposition [10,11]. However, previous studies mostly evaluated total upper airway deposition and have not distinguished deposition in the oropharyngeal and laryngeal regions. This distinction could be important. For instance, laryngeal deposition of inhaled corticosteroids (ICS), a mainstay in the treatment of chronic reactive airway disease, elicits local side effects, including dysphonia [12,13], that could be minimized with optimized flow rate for specific upper airway anatomy.

Understanding the mechanics of particle deposition in the upper airway is useful to design new inhalation therapies for respiratory diseases [14]. It also helps estimate exposure risks to inhaled toxicants [15] or airborne transmission of SARS-CoV-2-laden droplets [16]. Computational fluid particle dynamics (CFPD) has been used as a reliable method to predict airflow and particle deposition in the human extrathoracic airways [17,18,19]. In this study, we have undertaken CFPD studies to characterize the deposition of micrometer-sized particles (1–30 µm) at subject-specific inhalation flow rates in realistic geometries of the upper airway reconstructed from computed tomography (CT) scans of 11 individuals (7 healthy subjects and 4 mild-to-moderate COPD) for which subject-specific flow patterns and aerosol deposition data are also available [20]. Intersubject variability in shape and volume of the upper airway is expected to have a significant impact on the deposition of inhaled aerosols filtered by this region.

## 2. Materials and Methods

### 2.1. CT Database and Subject Characteristics

**Figure A1:**
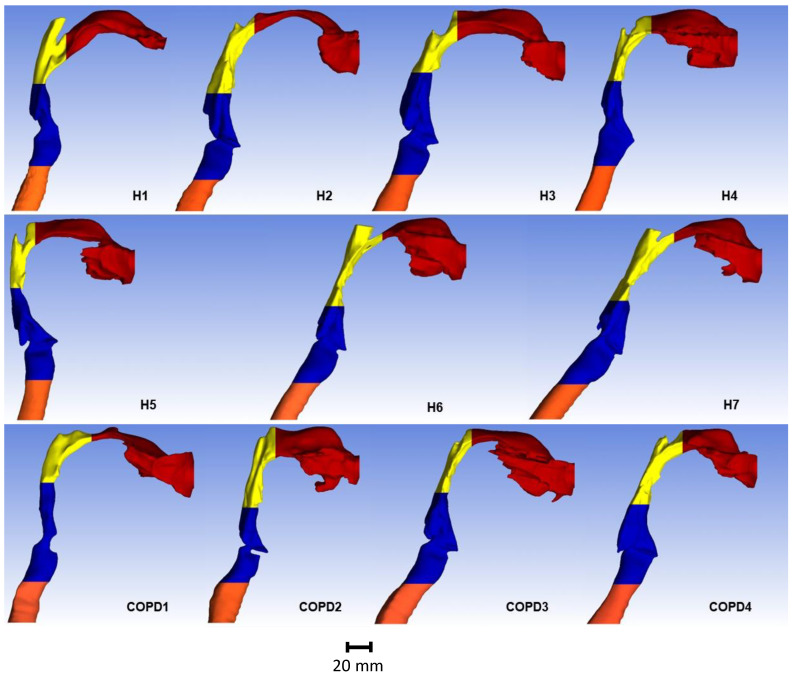
Upper airway geometry of all individuals. The same scale is used in all models.

The 3D upper airway models were based on CT images from seven healthy male subjects and four male subjects with mild-to-moderate chronic obstructive pulmonary disease (COPD). Images were previously obtained on a GE Light Speed Discovery CT750 and acquired as part of a Bioengineering Research Partnership (NIH R01-HL-073598). Images of the head and torso were obtained in the supine position during a breath hold at a lung volume of 1 L above functional residual capacity (FRC). CT scans were obtained with the mouthpiece positioned in the patient’s mouth. The field of view was 36 × 36 × 48 cm in the x, y and z dimensions (with the z-axis in the cranial to caudal direction). The matrix size was 512 × 512 × 960, generating voxel dimensions of 0.7 × 0.7 × 0.5 mm. Acquisition and use of these images were approved by the Institutional Review Boards of the University of Washington, Seattle and the University of California, San Diego, respectively. Anthropologic and lung function data of all subjects are presented in Table 1. Geometric data of all subjects are presented in Table A1 (see Appendix A) along with the 3D reconstructed geometry of the eleven subjects (Figure A1). There was no significant difference in the volume and surface area of the upper airway between healthy and COPD subjects.

### 2.2. Reconstruction of Human Upper Airway Geometries

**Figure 1:**
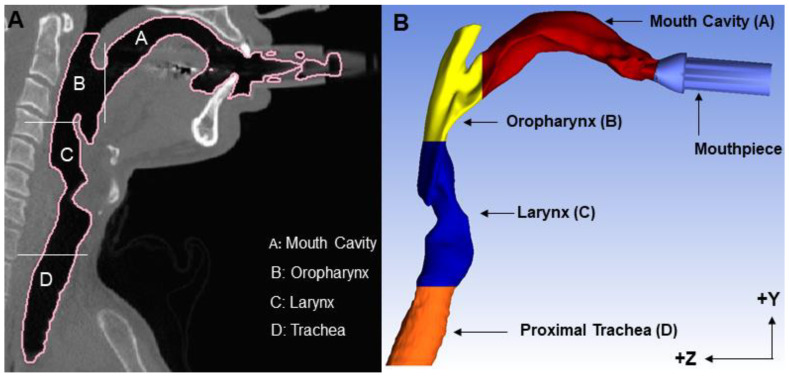
Definition of the subregions of the upper airway (**A**) CT scan of the oral airway of subject H1. (**B**) Reconstructed 3D model. The mouth is the oral cavity that extends from the back of the teeth to the uvula. The oropharynx region is the cavity from the back of the uvula to the tip of epiglottis. The larynx region is from the tip of the epiglottis to below the vocal cords and top of the trachea. The Z direction indicates the direction of gravity.

CT scans in DICOM format were used to create three-dimensional (3D) realistic models of the oral airway anatomy using Mimics 23.0 (Materialise Inc., Leuven, Belgium). Briefly, by setting a previously identified optimal threshold between −1024 and −300 Hounsfield Units (HU) [21,22], the 3D anatomy of the oral cavity, including the mouth, oropharyngeal region, larynx, vocal cord, and upper tracheal sections, was selected as the region of interest (ROI). The nasal cavity and paranasal sinuses were excluded. The oral airway geometries were exported in stereolithography (stl) format to ICEM-CFD 21.0 (ANSYS 2021 R2, Inc., Canonsburg, PA, USA), where the planar inlet and outlet surfaces were defined. All geometries were oriented with the oral cavity floor parallel to the Z-axis (Figure 1). Planar divisions between the posterior mouth, oropharynx, area of larynx and trachea were created for recording region-specific particle deposition in airflow simulations. Consistent with experimental conditions, a 245 mm-long tube was added to the mouthpiece.

### 2.3. Computational Fluid-Particle Dynamics (CFPD) Simulations

#### 2.3.1. Flow Simulations

CFPD simulations were performed by solving Navier–Stokes equations with Lagrangian particle tracking. The volume fraction of the particles in the air was in the range of 10^−7^ to 10^−9^. Particle number density was thus sufficiently low so that particle motion did not affect airflow and a one-way coupling between airflow and particle transport was used [23]. The Navier–Stokes equation for incompressible flow is

(1) $$\rho \frac{\partial u}{\partial t}+\rho \left(u.\nabla \right)u=-\nabla p+\mu \nabla^{2}\;u$$

where u=u(x,y,z,t) is the fluid velocity vector, ρ=1.204 kg/m^3^ is the fluid density, $\mu =1.825\times 10^{-5}$ kg/ (m.s) is the dynamic viscosity of the Newtonian fluid (i.e., air), p is pressure and t is time. To solve the Navier–Stokes equation, mesh independence analysis has been performed, and geometries were meshed in ICEM-CFD 21.0, a preprocessor tool from Ansys Fluent, with approximately twelve million tetrahedral elements containing eight prism layers with a total prism zone thickness of 0.2 mm. Prism mesh was generated near the wall to ensure capturing of the steep air velocity gradient and of accurate particle deposition patterns in the simulations. Inhalation rates representative of resting and fast breathing were considered. Mesh quality was checked by considering the quality criteria of greater than 0.1 to ensure that the mesh had no distorted elements [24].

Experimental evidence suggests that flow in the extrathoracic airway is laminar at resting breathing rates [25,26]. Thus, for slow-breathing condition (18 L/min), Equation (1) was solved in the steady-state laminar flow regime by employing Ansys Fluent 2021 R2 (ANSYS, Inc., Canonsburg, PA, USA) and choosing SIMPLEC algorithm for pressure-velocity coupling and second-order upwind discretization method. Steady-state flow simulations were conducted by imposing boundary conditions on the inlet mass flow rate of the air and pressure outlet boundary conditions applied at the outlet boundary. To ensure numerically accurate flow simulation results, the Shear-Stress Transport *k*-ω turbulent model (SST *k*-ω) was employed to simulate the inspiratory and expiratory airflow for fast-breathing conditions (45 L/min) [27]. The turbulence length scale was considered 0.001 m, and turbulent intensity was assumed to be 5% at the inlet and outlet [28,29].

The following boundary conditions for the flow were applied: (1) zero velocity at the walls (no-slip boundary condition), (2) inlet mass flow at the mouthpiece’s inlet to set airflow of 18 and 45 L/min, and (3) zero pressure at the outlet.

#### 2.3.2. Particle Transport Simulations

Forces affecting particles for the size range used in this study included drag force and gravity (consistent with experimental orientation), allowing modeling of deposition mechanisms of inertial impaction and sedimentation. The equation of motion governing the trajectory of a particle is

(2) $$\frac{du_{p}}{dt}=F_{D}\left(u-u_{p}\right)+\frac{g\left(\rho_{p\;-\;}\rho \right)}{\rho_{p}}$$

where $u_{p}$ is the particle velocity, **g** is the gravity field, $\rho_{p}$ is the density of the particle, $F_{D}\left(u-u_{p}\right)$ is the drag force per unit particle mass and

(3) $$F_{D}=\frac{18\mu }{\rho_{p}d_{p}^{2}}\frac{C_{D}Re_{p}}{24}$$

where $d_{p}$ is the particle diameter, *Re_p_*, the particle Reynolds number and $C_{D}$ is the drag coefficient [24].

The Lagrangian-based Discrete Phase Model (DPM) was used to predict particle deposition in the eleven anatomically realistic models. Deposition of particles with mass median aerodynamic diameters (MMAD) of 1–30 µm (1 µm increments) was investigated during resting and fast-breathing conditions. Particles were considered inert with a spherical shape and density of 1000 kg/m³ so that the particle diameter corresponds to the aerodynamic diameter. Particles were released from the inlet of the tube. As the tube and mouthpiece were not in the region of interest, the boundary condition “reflect” was applied in the DPM simulation. Boundary condition “trap” was considered for the mouth cavity, oropharyngeal, laryngeal, and tracheal region to predict aerosol deposition. The “escape” boundary condition was used at the outlet. The trajectories of 10,000 particles were simulated for each particle size and inhalation flow rate, allowing for particle number-independent predictions. Particles were injected at the inlet with a blunt profile and random spatial distribution. Increasing to 100,000 particles changed predicted deposition by less than 0.38%.

The percentages of inhaled particles deposited in each anatomical region (Figure 1) were quantified. For instance, particle deposition in the laryngeal region was defined as 100 × (N_L_/N_I_), where N_I_ is the total number of particles inhaled and N_L_ is the number of particles deposited on the surface region mapped as the laryngeal region on the airway model. Similarly, the total deposition efficiency was defined as 100 × (N_T_/N_I_), where N_T_ is the total number of particles deposited in all airway regions combined.

### 2.4. Comparison of Subject-Specific In Silico Predictions with Experimental Results

Whole-lung deposition predictions were obtained for breathing parameters and functional residual capacity (FRC) that matched on a subject-by-subject basis those measured in 7 healthy subjects during controlled breathing of aerosols [20]. In these experiments, subjects were asked to target a tidal volume of 1000 mL of particle-laden air (1 and 2.9 µm) at constant inhalation flow rates of 18 and 45 L/min. Actual tidal volumes and flow rates measured over five consecutive breaths of controlled exposure for each experimental condition are listed in Table 2 along with each subject FRC. These data were used in the in silico predictions used in the comparison.

The overall retained fraction was calculated as the sum of the deposition fraction in the oral cavity and retained fraction in the intrathoracic lung. The intrathoracic deposition was estimated with the latest version of the Multiple-Path Particle Dosimetry (MPPD) model that includes a mechanistically based model component for alveolar mixing of particles and that accounts for multiple breaths of aerosol intake [30]. Deposition in the oral cavity was obtained from CFD simulations as described above. Deposition in the oral cavity was also obtained from the semi-empirical equation of Stahlhofen et al. [31]:

(4) $$\eta_{oral}=1-{\left(3.5\times 10^{-8}\left(d_{a}^{2}Q\right){\;}^{1.7}+1\right)}^{-1}$$

where *d_a_* is the aerodynamic diameter expressed in μm and *Q* is the overall volumetric flow rate expressed in mL/s.

The mass of particles injected in the MPPD model was set as (1 − *η_oral_*) × *C_inh_* where *C_inh_* is the inhaled particle concentration. Deposition in the oral cavity during exhalation was based on the mass of particles exiting MPPD (*η_oral_ C_exh.MPPD_*).

### 2.5. Statistical Analysis

The curve of best fit of in silico predictions of aerosol deposition in the oral cavity was calculated by fitting $\beta_{1}$ and $\beta_{2}$ to a sigmoidal function $DE=1-{\left(\beta_{1}{\left(d_{a}^{2}Q\right)}^{\beta_{2}}+1\right)}^{-1}$, where $d_{a}^{2}Q$ is the same impaction parameter used in the Stahlhofen equation (Equation (4)). $\beta_{1}$, $\beta_{2}$, and the percentage of variance explained ($R^{2}$) by the curve of best fit were compared to the parameters adapted in the Stahlhofen equation.

To compare in silico predictions with experimental data, a one-way ANOVA test for correlated samples was used with the Tukey Multiple Comparison post hoc test. The paired t test was used to compare in silico predictions of oral deposition between inspiration and expiration. Significant differences were accepted at the *p* < 0.05 level.

MATLAB R2022a was used for curve fitting and confidence interval estimation, and R v4.0.2 was used for statistical analysis and visualization.

## 3. Results

### 3.1. Total Oral Deposition Efficiency

**Figure 2:**
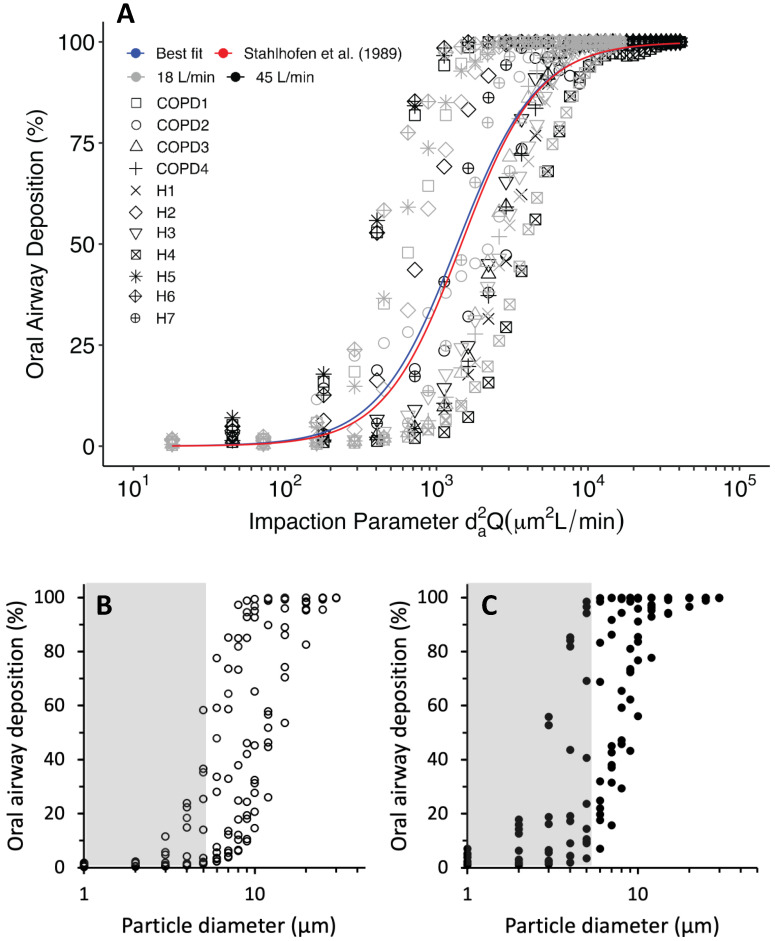
Oral airway deposition. (**A**): Oral airway deposition vs. inertial impaction parameter. Gray symbols and black symbols represent deposition fraction at inhalation flowrate 18 and 45 L/min, respectively. Empirical prediction is based on Stahlhofen equation (Equation (4)). (**B**,**C**): Deposition vs. particle diameter at an inhalation flow rate of 18 L/min (panel (**B**)) and 45 L/min (panel (**C**)). For particles >10 µm in diameter, data are only shown for 12, 15, 20, 25 and 30 µm. Gray area in each panel highlights the optimum aerodynamic particle size distribution (i.e., 1–5 µm) for most pharmaceutical aerosols.

Figure 2A illustrates oral deposition efficiency as a function of the commonly used impaction parameter *d_a_*^2^*Q*, where *d_a_* (µm) is the aerodynamic diameter and *Q* (L/min) is the flow rate. In agreement with previous in vitro and in silico studies [8,9,18,32], the high correlation (Figure 2A, best fit, R^2^ = 86.02%) between oral deposition and the impaction parameter *d_a_*^2^*Q* shows that inertial impaction is the dominant deposition mechanism in the upper airway. Total oral deposition of inhaled particles increased with increasing particle size and inhalation flow rate. For instance, in subject H1 and for a flow rate of 18 L/min, oral deposition increases from 0.26% for 1 µm particles (gray × symbol at *d_a_*^2^*Q* = 18 µm^2^L/min, Figure 2A) to 1.49% for 5µm particles (gray × symbol at *d_a_*^2^*Q* = 450 µm^2^L/min) and to 20.71% for 10 µm particles (gray × symbol at *d_a_*^2^*Q* = 1800 µm^2^L/min). In other words, 99.74% of 1 µm particles, 98.51% of 5µm particles and 79.29% of 10 µm particles traversed the airway model and were delivered to the intrathoracic region of the lung. Increasing the inhalation rate caused a substantial increase in the percentage of particles deposited in the upper airway. Simulations for a 45 L/min inhalation rate predicted 1.21% of 1 µm particles (black × symbol at *d_a_*^2^*Q* = 45 µm^2^L/min, Figure 2A) and 76.78% of 10 µm particles (black × symbol at *d_a_*^2^*Q* = 4500 µm^2^L/min) depositing in the upper airway model, respectively. While most of 1 µm particles were delivered to the trachea at both flow rates, <25% of inhaled 10 µm particles were delivered to the intrathoracic lungs at the higher flow rate compared to ~80% at the low flow rate. Despite larger intrasubject variability in oral deposition, similar trends were observed for all subjects (Figure 2). For instance, deposition of 3 µm aerosols ranged from 0.51% to 11.59% (median = 0.97%) between subjects at 18 L/min (Figure 2B) and from 0.84% to 52.32% (median 6.24%) at 45 L/min (Figure 2C). There was no significant difference in oral airway deposition between healthy and mild-to-moderate COPD subjects.

Predictions of oral deposition in both healthy and COPD cohorts show a good agreement with the empirical curve previously obtained by Stahlhofen et al. [31] from controlled in vivo experiments (red curve, Figure 2A). The equation of best fit to patient-specific CFD data was [1 − (1/(*a*(*d_a_*^2^*Q*)^*b*^ + 1)], where *a* = 6.73 × 10^−8^ (−7.33 × 10^−9^, 1.42 × 10^−7^), *b* = 1.65 (1.55, 1.74) (R^2^*_best fit_* = 0.8602).

### 3.2. Effect of Particle Impaction on Regional Deposition

**Figure 3:**
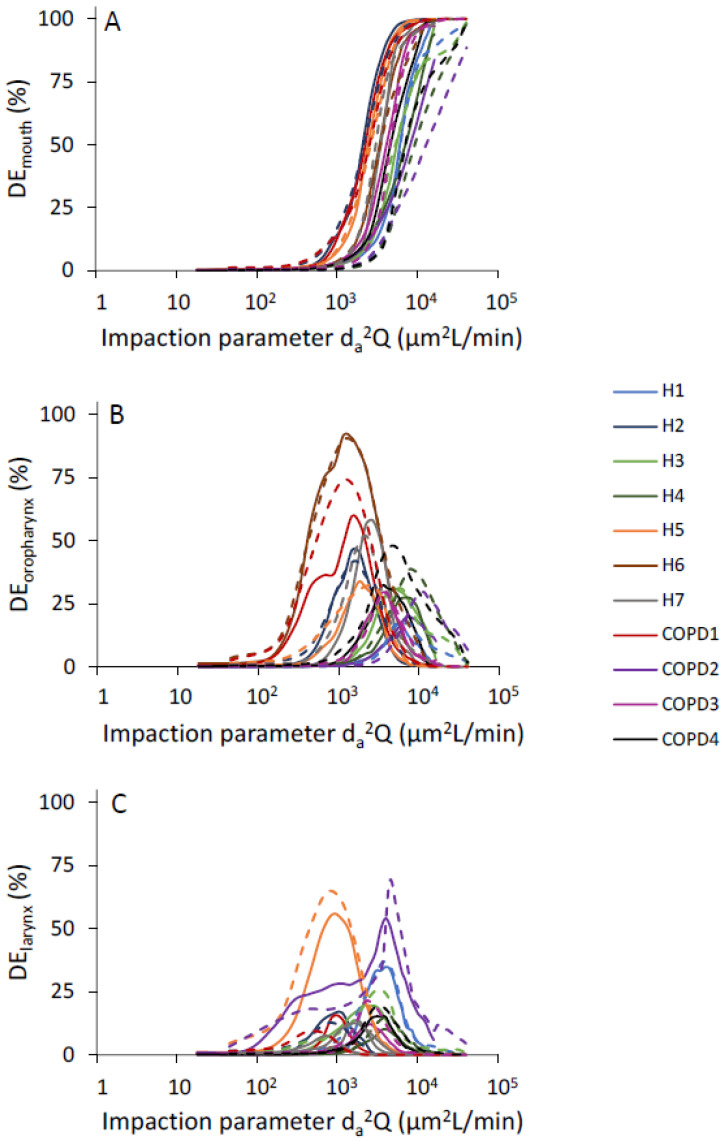
Inspiratory regional deposition (DE) vs. inertial impaction parameter. Solid and dashed lines represent deposition fraction at inhalation flow rate of 18 (L/min) and 45 (L/min), respectively. (**A**): Mouth. (**B**): Oropharynx. (**C**): Larynx.

Regional deposition fractions were computed as a function of *d_a_*^2^*Q* for all the subregions of the upper airway as defined in Figure 1. There was significant intersubject variability in the distribution of deposited particles among all three subregions of the upper airway (Figure 3A–C). Deposition in the mouth cavity increased with increasing particle size and increasing flow rate (Figure 3A). Deposition in both the oropharyngeal and the laryngeal region followed a bell shape, with the maximum deposition varying largely between subjects: maximum deposition occurred for an impaction parameter ranging between 10^3^ and 1.2 × 10^4^ in the oropharyngeal region (Figure 3B) and between 400 and 5000 in the laryngeal region (Figure 3C).

### 3.3. Comparison of Inspiratory and Expiratory Particle Deposition in the Upper Airway

**Figure 4:**
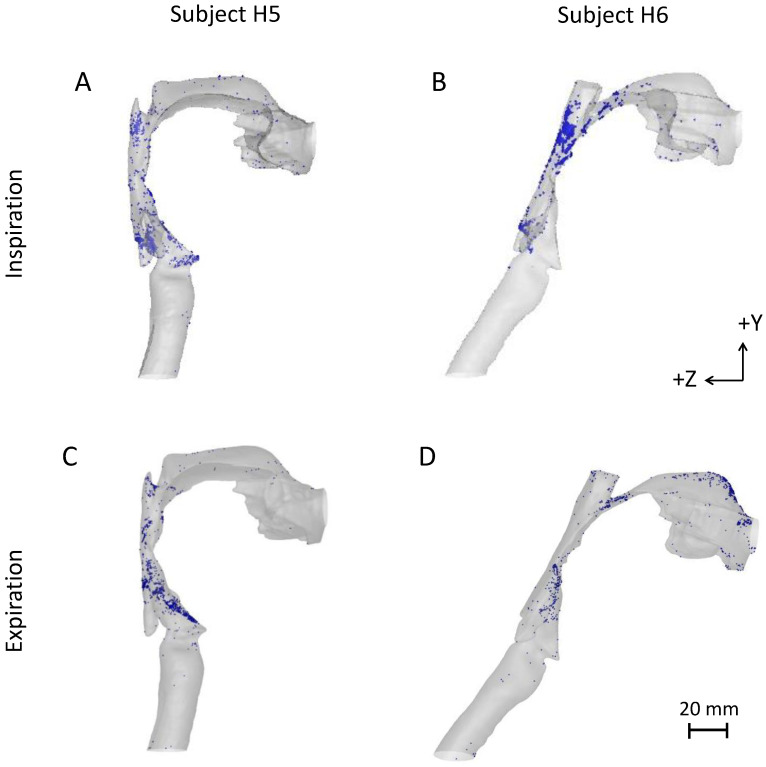
Spatial distribution of deposited 3 µm particles in two subjects with largely different upper airway anatomy. Data are shown for an inspiratory (**A**,**B**) and expiratory (**C**,**D**) flow rate of 45 L/min in subject H5 (**A**,**C**) and H6 (**B**,**D**).

**Figure A2:**
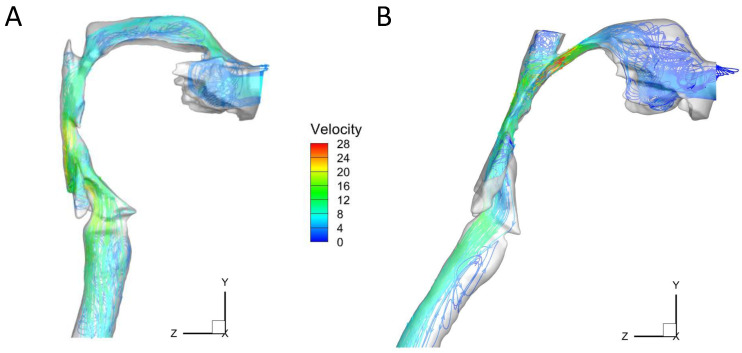
Velocity streamlines in the upper airway of (**A**) subject H5 and (**B**) subject H6 during inhalation at a flow rate of 45 L/min. Velocity scale is shown in m/s.

The effect of flow direction (inspiratory versus expiratory flow) on oral deposition was also investigated. For expiratory flow simulations, particles were injected at the tracheal outlet with a blunt profile and random spatial distribution. Figure 4 shows the spatial distribution of deposited particles in two subjects with highly different upper airway shapes following inhalation or exhalation of 3 µm particles. In subject H5, hotspots of deposited particles were mainly found on the posterior laryngeal wall and at the level of the vocal cords following inhalation (Figure 4A) and on the anterior laryngeal wall following exhalation (Figure 4C). In contrast, in subject H6, most deposited particles were located on the posterior oropharyngeal wall following inhalation (Figure 4B) and on the anterior laryngeal wall and on the hard palate (mouth cavity) following exhalation (Figure 4D). The hotspots were associated with regions of high airflow velocities (Figure A2), again suggesting that inertial impaction is the dominant deposition mechanism in the upper airway. These data not only highlight the large variability in deposition patterns between subjects but also between breathing phases, i.e., between inspiration and expiration.

**Figure 5:**
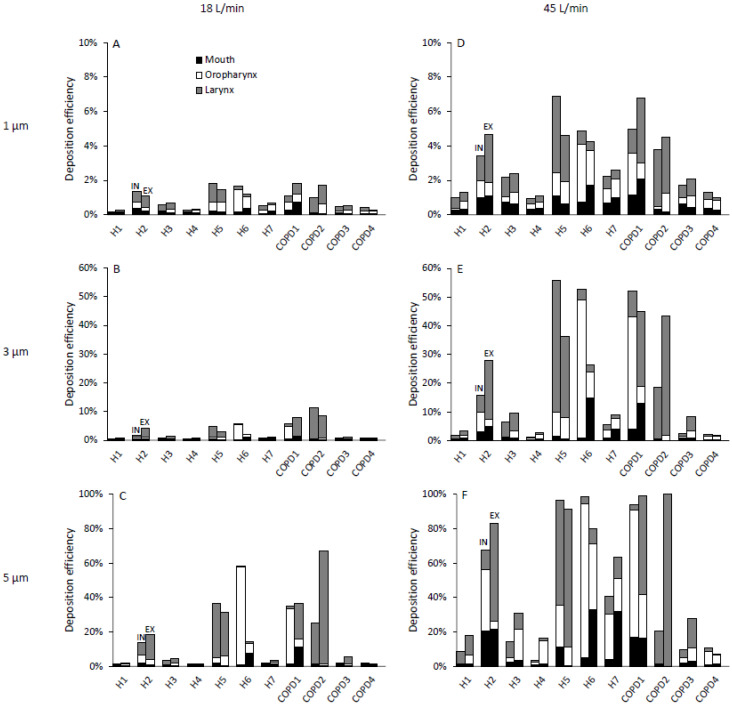
Comparison of deposition occurring during inhalation (IN) and exhalation (EX) in the different subregions of the oral airway. Deposition for 1, 3 and 5 µm particles are shown in top (**A**,**D**), middle (**B**,**E**) and lower panels (**C**,**F**), respectively. Deposition at a flow rate of 18 L/min and 45 L/min are shown in the left (**A**–**C**) and right panels (**D**–**F**), respectively. Note the y-axis range differs for each particle size.

**Figure 6:**
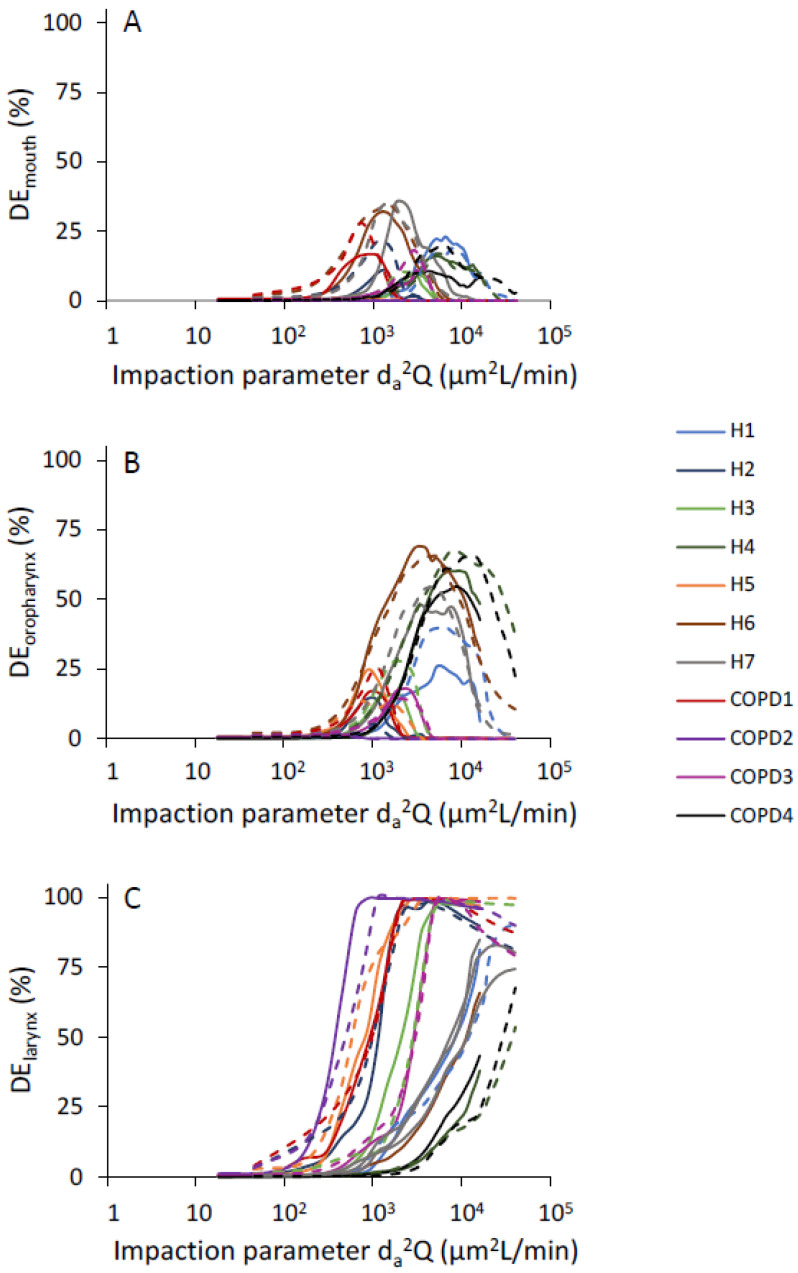
Expiratory regional deposition (DE) vs. inertial impaction parameter. Solid and dashed lines represent deposition fraction at exhalation flowrate of 18 (L/min) and 45 (L/min), respectively. (**A**): Mouth. (**B**): Oropharynx. (**C**): Larynx.

Distribution of 1, 3 and 5 µm deposited particles at 18 L/min and 45 L/min breathing conditions among subregions of the subject-specific oral airway is shown for all subjects in Figure 5 both for inhalation and exhalation. These CFD results do not show any consistent trend when deposition occurring during inhalation is compared to that during expiration, with some subjects showing higher deposition during inhalation, others showing higher deposition during exhalation and a third group showing similar values between inspiration and expiration. As a result, there was no significant difference in oral deposition between inspiration and expiration in this group of subjects. This was also true for subregion deposition except for particles ≥ 5 µm (Figure 5F) where most particles deposited in the laryngeal region during expiration, leaving few particles to travel and potentially deposit in the oropharynx and mouth cavity. Regional deposition for all particle sizes (1–30 µm) and expiratory flow rate (18 and 45 L/min) is shown in Figure 6. Deposition in the laryngeal region increased with increasing particle size and increasing expiratory flow rate (Figure 6C), while deposition in both the oropharyngeal region and the mouth cavity followed a bell shape, with the maximum deposition varying largely between subjects (Figure 6A,B).

### 3.4. Comparison of In Silico Predictions with In Vivo Experimental Data

**Figure 7:**
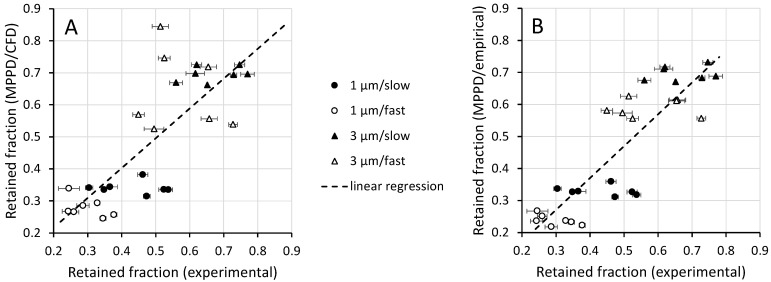
Comparison of in silico predictions with experimental data. Upper airway deposition was predicted by CFD (panel (**A**)) or calculated from Equation (4) (panel (**B**)). Retained fraction in the intrathoracic lung was obtained from the Multiple-Path Particle Model (MPPD) in both cases. Dashed line: regression line between experimental and predicted retained fraction (R^2^ = 61% in panel (**A**) and 73% in panel (**B**)). See text for details.

**Figure 8:**
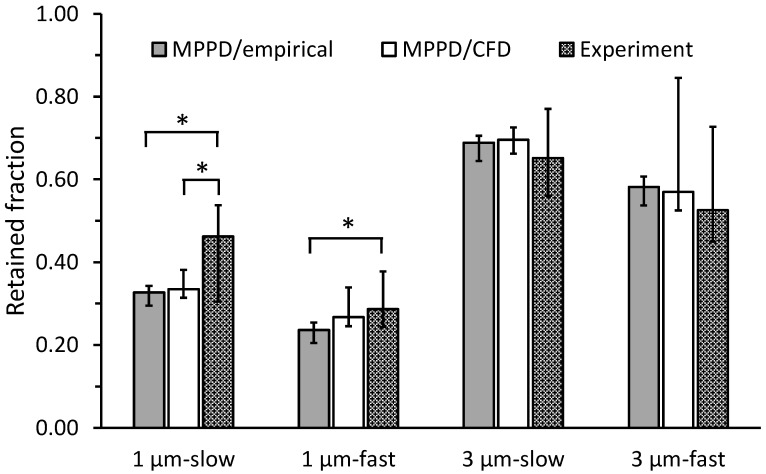
Comparison of retained fraction predicted by the MPPD/empirical and MPPD/CFD models with measurements of Darquenne et al. [20]. In silico predictions were obtained for subject-specific breathing conditions matching the experiments. Data are shown as the median (minimum, maximum) of the 7 healthy subjects. *: significantly different from experiments (*p* < 0.02). See text for details.

Whole-lung deposition predictions were obtained by coupling subject-specific CFD results with MPPD predictions as described in Section 2.4. Figure 7A displays these predictions against experimental data obtained by Darquenne et al. [20]. Experimental data are displayed as deposition measured over five consecutive breaths (mean ± standard deviation (SD)). Whole-lung deposition was also obtained by coupling predictions from Equation (4) with MPPD (Figure 7B). The regression line between in silico predictions (y) and experimental data (x) were y = 0.931x + 0.030; R^2^ = 61%, for MPPD/CFD results (Figure 7A, dashed line) and y = 0.995x − 0.028; R^2^ = 73%, for MPPD/empirical results (Figure 7B). Figure 8 compares whole-lung deposition predictions for the seven subjects grouped together for both the MPPD/CFD and MPPD/empirical cases and the experimental data. Data are presented as median (minimum, maximum).

## 4. Discussion

### 4.1. Intersubject Variability in Deposition in the Oral Airway

Oral deposition of particles in the aerodynamic size range of 1–30 µm was numerically predicted in distinct geometries of oral airways of seven healthy adults and four subjects with mild-to-moderate COPD. Lung diseases such as COPD significantly alter the deposition of inhaled particles in the intrathoracic lungs [33]. This is mainly because of alterations in the geometry of the airways and alveolar spaces that result in regional changes in the ventilation distribution of inhaled air and flow patterns in the airspaces [34]. To our knowledge, there is no report of significant alterations in the geometry of the extrathoracic airway between healthy subjects and those with mild-to-moderate COPD. For similar exposure conditions, we found no significant difference in oral airway deposition between healthy and mild-to-moderate COPD subjects.

For any given combination of particle size and inhaled flow rate, a large scatter was observed between subjects. For example, deposition ranged from 0–18%, 1–56% and 2–85% at an impact parameter of ~200, 400 and 1000 µm^2^ L/min, respectively (Figure 2A). These data compare well with in vitro measurements obtained in nine replicas of oral airways where deposition ranged from 0–30%, 0–60% and 5–95% at an impact parameter of 200, 400 and 1000 µ^2^ L/min, respectively [9]. A similar scatter in oral deposition between subjects was also observed by Grgic et al. in a separate in vitro study [8]. 

Data from in vivo studies have also reported large intersubject variability [35,36,37,38,39,40,41]. Using these in vivo data, Stahlhofen et al. derived a semi-empirical equation based on particle size and breathing pattern characterized by the impaction parameter (Equation (4)). We developed a similar correlation based on our in silico predictions (Figure 2A). Despite pronounced intersubject variability in deposition predictions, the sigmoidal curve of in silico oral deposition versus impaction parameter remains statistically indifferent from the experimental curve predicted by the Stahlhofen equation, which alone explains the experimental data well with a best fit R^2^ value of 85.98%. This compares to an R^2^ of 86.02% for the sigmoidal curve based on in silico predictions.

The flow rates used in this study are representative of those achieved during aerosol delivered by nebulizers (18 L/min, tidal breathing) and dry powder inhalers (DPIs, 45 L/min, target 30–60 L/min). With an optimal size distribution of 1–5 µm for pharmaceutical aerosols, our data suggest that, with a nebulizer, more than 75% of the inhaled aerosol would be delivered to the intrathoracic lungs in most subjects (gray area, Figure 2B), with only a few of them having >25% of the larger particles (i.e., 5 µm) depositing in the upper airway. In contrast, there is a much larger variability with the use of a DPI with only about half of the subjects delivering >75% of the 1–5 µm aerosol to the intrathoracic lungs (gray area, Figure 2C). These data show that a more consistent aerosol dose among subjects can be delivered to the intrathoracic lungs when using nebulizers (that require a lower flow rate) than DPIs (that require a higher flow rate). In terms of in silico population studies of aerosol deposition in the upper airway and tracheobronchial trees, these data also suggest that, for aerosol exposure conditions that resulted in low inter-subject variability, a standardized oral upper airway model could be used, resulting in significant computational time savings [42].

Fewer studies have looked at regional deposition within the oral extrathoracic airway [8,43] but it is often assumed that, during inhalation, most deposition occurs at the level of sudden constrictions present in the oropharyngeal and laryngeal regions [31,38]. Our data suggest this to be the case for impaction parameters up to ~1000–2000 µm^2^ L/min. However, for larger values of the impaction parameter, deposition in the mouth also becomes significant (Figure 3A). As a result, deposition in the oropharynx resembles a bell-shaped curve (Figure 3B) rather than the sigmoid shape seen in the mouth. A similar bell-shaped curve was obtained in the larynx (Figure 3C). Compared to deposition in the oropharynx, the curve was however further shifted towards the smaller particles due to additional upstream deposition. Finally, a comparison of our in silico predictions with in vitro data obtained in seven realistic mouth-throat geometries for impaction parameters ranging from 270 to 3800 µm^2^ L/min shows relatively good agreement [8]. Indeed, these in vitro data not only demonstrated high intersubject variability in the regional deposition but also significant deposition in the mouth for experiments performed with impaction parameters around 1000 µm^2^ L/min or larger.

### 4.2. Differences in Regional Deposition between Inhalation and Exhalation

Most of the studies on upper airway deposition during mouth breathing have focused on the inspiratory phase, e.g [2,5,8,9,32], with very few looking at a deposition during expiration [7,43]. This is mainly because inhalation drug therapies are designed to maximize deposition in the lungs prior to exhalation. Indeed, when using a dry powder inhaler (DPI) or a pressurized metered-dose inhaler (pMDI), proper drug inhalation techniques call for a deep inhalation followed by a breath hold to allow for particles to settle in the airspaces, minimizing the number of particles being exhaled [44]. However, poor inhalation technique can result in a significant fraction of exhaled aerosol. Additionally, drugs administered with nebulizers do not typically include an end-inspiratory breath-hold, which can result in significant exhaled aerosol fractions.

Due to the paucity of available data for the exhalation mode, some authors have suggested that deposition in the oral cavity during exhalation could be neglected [31] while others have proposed that inspiratory and expiratory deposition efficiency in the oral cavity should be considered to be equal [7,45]. Our data suggest the latter to be a reasonable hypothesis, at least for micron-sized particles. Indeed, there was no significant difference between inspiratory and expiratory deposition in the oral cavity for this size range (Figure 5). This is also supported by data from Verbanck et al. [43], albeit performed in a single oral airway geometry, that showed almost identical oral deposition curves for inspiration and expiration for experiments performed with 3 and 6 µm particles.

In terms of regional deposition, intrasubject differences were observed when the airflow direction was reversed. While during inspiration, hot spots were mainly found in the oropharyngeal region and also in the mouth for the largest particles (10 µm and up), high deposition was preferentially found in the laryngeal region during expiration with minimal deposition in the mouth cavity for large values of $d_{a}^{2}Q$ (Figure 4 and Figure 6). When averaged over all subjects, these differences were statistically significant for particles ≥ 5 µm. This may be of minimal clinical relevance for the largest particles (>20 µm) that tend to have high intrathoracic deposition rates, leaving only a negligible particle fraction, if any, to be exhaled. In contrast, different deposition patterns may be of importance when assessing the side effects of an inhaled drug with particle size distribution in the range of 1–10 µm, typical of most pharmaceutical aerosols. For example, the delivery of inhaled corticosteroids (ICS) is highly effective at controlling the inflammatory component of chronic airway disease such as in asthma and COPD, while limiting systemic toxicities [46]. However, the efficacy of ICS is highly dependent upon its ability to bypass the upper airway. High extrathoracic deposition not only limits the amount of drug that can reach the lungs but can also result in unwanted local side effects. Indeed, the repeated deposition of corticosteroids in the larynx can cause a wide variety of clinical side effects including hoarseness, sore and/or dry throat, dysphonia, and candidiasis [12,47].

### 4.3. Comparison of Whole-Lung Deposition with Experiments

In an attempt to reproduce aerosol exposure studies performed on the same subjects from which the upper airway geometries were obtained, we predicted deposition for breathing maneuvers similar to that used in the experiments [20]. To do so, we coupled our predictions of oral deposition to an intrathoracic deposition obtained with an improved MPPD model [30] that also accounted for subject-specific lung volumes and subject-specific inhalation and exhalation flow rates (Table 2). As the MPPD model assumes uniform ventilation among the different regions of the lung, we limited our comparison to whole-lung deposition data obtained in healthy subjects for which, unlike in COPD subjects, a uniform ventilation distribution is a reasonable assumption. Comparison of our MPPD/CFD predictions with experimental values shows relatively good agreement (Figure 7A) as did the comparison between experimental data and MPPD predictions coupled with the Stahlhofen equation for oral deposition (MPPD/empirical predictions, Figure 7B). As the Stahlhofen equation only incorporates particle size and flow rate characteristics, less scatter was observed in the MPPD/empirical predictions than in the MPPD/CFD data, the latter also reflecting the effect of upper airway geometry on oral deposition (Figure 8).

There are a few limitations worth noting that could have affected our predictions. First, CFD simulations were performed in upper airway geometries with rigid walls that were based on CT images obtained at the end of a one-liter inspiration. Thus, the effect of any variation in the upper airway geometry that occurs during tidal breathing even in healthy subjects with no upper airway pathology [48,49] was neglected. Second, as CT imaging and aerosol studies were performed on two different occasions, it is highly probable that the position of the tongue within the oral cavity changed between the sessions. Furthermore, the position of the tongue was not controlled for during the aerosol studies and its position may well have moved during the experiment. This would affect deposition predictions in the oral cavity. Indeed, previous studies have shown that the position of the tongue significantly affects the delivery of aerosol in the trachea, highlighting the high intrasubject variability in aerosol delivery to the lungs [50,51,52].

## 5. Conclusions

Drug inhalation is a mainstay in the management of respiratory diseases. Its success does not only depend on the pharmacology of the drugs being inhaled but also on the site and extent of deposition in the respiratory tract. Both the physical properties of the inhaled aerosols and the subject characteristics (i.e., lung volume and geometry, breathing pattern, disease) strongly affect deposition. The upper airway is characterized by variable cross-sectional areas and sharp changes in a flow direction that is conducive to deposition by inertial impaction. Most pharmaceutical aerosols target the intrathoracic lung and as such, deposition in the oral passages should be kept to a minimum to circumvent local adverse effects and to maximize intrathoracic lung dose.

Deposition of 1–30 µm particles was predicted in eleven models of oral airways of adults at two flow rates, one flow typical of tidal breathing/nebulizer use and one flow in the range recommended when using a DPI. For an optimal size distribution of 1–5 µm for pharmaceutical aerosols, our data suggest that >75% of the inhaled aerosol is delivered to the intrathoracic lungs in most subjects when using a nebulizer but only in about half the subjects when using a DPI. This is also the first report of in silico predictions in such many geometries for both inhalation and exhalation. Averaged over all geometries, our data showed no significant difference in overall deposition efficiency in the oral cavity between the inspiratory and expiratory phases. In contrast, subregional patterns largely differed between the two phases, with areas of hot spots preferentially in the oropharynx during inspiration, and in the laryngeal region during expiration.

## Figures and Tables

**Table 1:** Anthropometric data.

Subject ID	Age, Yr	Weight, kg	Height, cm	BMI	Health Status	FEV_1_, %pred	FEV_1_/FVC
H1	35	68.2	170	23.5	Healthy	113	0.88
H2	52	96.8	165	35.5	Healthy	117	0.79
H3	47	88.6	183	26.5	Healthy	85	0.74
H4	26	91.8	183	27.5	Healthy	94	0.80
H5	34	100.0	193	26.8	Healthy	104	0.84
H6	21	54.1	168	19.2	Healthy	89	0.73
H7	21	63.6	173	21.3	Healthy	95	0.81
COPD1	57	70.0	164	26.1	COPD	60	0.56
COPD2	55	65.9	178	20.8	COPD	56	0.48
COPD3	45	83.2	180	25.6	COPD	69	0.67
COPD4	54	83.6	187	24.0	COPD	58	0.52

FEV_1_, forced expiratory volume in 1 s; FVC, forced vital capacity, %pred: % predicted.

**Table 2:** Patient-specific flow rates in healthy subjects.

		Subject	H1	H2	H3	H4	H5	H6	H7
		**FRC (L)**	3.26	3.38	3.44	3.51	2.67	3.43	3.31
**d_p_ = 1 µm**	**slow** **breathing**	**Q_in_ (L/min)**	19.26	19.08	19.86	17.70	18.72	17.64	18.18
		**Q_ex_ (L/min)**	20.94	19.32	20.10	18.24	18.66	17.82	17.76
		**TV (L)**	1.116	1.073	1.101	0.979	1.041	0.984	1.009
	**fast** **breathing**	**Q_in_ (L/min)**	43.56	41.40	41.94	39.18	40.56	42.90	41.16
		**Q_ex_ (L/min)**	45.30	40.02	41.64	40.50	42.18	40.02	40.74
		**TV (L)**	1.127	1.019	1.038	1.012	1.043	0.924	0.923
**d_p_ = 2.9 µm**	**slow** **breathing**	**Q_in_ (L/min)**	21.66	19.38	17.70	17.16	18.66	18.18	18.30
		**Q_ex_ (L/min)**	23.16	18.96	17.94	17.70	18.00	19.26	19.14
		**TV (L)**	1.254	1.064	0.987	0.972	1.023	1.043	1.249
	**fast** **breathing**	**Q_in_ (L/min)**	45.48	44.58	39.96	37.62	40.32	40.08	40.26
		**Q_ex_ (L/min)**	49.02	44.22	39.60	41.40	40.14	39.24	41.64
		**TV (L)**	1.164	1.135	0.994	0.993	1.035	0.900	0.901

d_p_: particle diameter, Q: flow rate, in: inspiration, ex: expiration, FRC: functional residual capacity, TV: tidal volume.

## Data Availability

The data presented in this study are available on reasonable request from the corresponding author.

## Appendix A. Individual Geometries

Table A1 lists the volume and surface area of each upper airway geometry shown in Figure A1.

**Table A1:** Dimension of the oral airways.

Subject ID	Volume (cm³)	Surface Area (cm²)
H1	73.70	188.18
H2	65.32	178.55
H3	63.88	179.94
H4	96.34	210.22
H5	67.92	193.32
H6	72.51	200.18
H7	61.28	192.96
COPD1	52.86	149.79
COPD2	126.83	254.02
COPD3	85.53	243.09
COPD4	87.40	220.84
